# Effect of Galacto-oligosaccharides with Different Degrees of Polymerization on the Storage Stability of Freeze-Dried Lecithin Liposomes

**DOI:** 10.3390/foods15152734

**Published:** 2026-08-04

**Authors:** Juan Manuel Faroux, Micaela Ureta, Andrea Gomez-Zavaglia, Emma E. Tymczszyn

**Affiliations:** 1 Center for Research and Development in Food Science and Technology (CIDCA, CCT-CONICET La Plata), La Plata RA 1900, Argentinamicaelaureta@gmail.com (M.U.); 2 Laboratorio de Microbiología Molecular, Instituto de Microbiología Básica y Aplicada (IMBA, CIC-PBA), Universidad Nacional de Quilmes, Bernal RA 1876, Argentina; elitym@yahoo.com.ar

**Keywords:** galacto-oligosaccharides, glass transition temperature, lipid oxidation, liposomes, freeze-drying

## Abstract

Galacto-oligosaccharides (GOS) are widely recognized as prebiotic ingredients, whereas their technological role in stabilizing lipid-based systems remains poorly understood. In this study, GOS were synthesized by enzymatic transgalactosylation of lactose and enriched by ethanol precipitation. Carbohydrate composition, glass transition temperature (*Tg*), and their influence on the formation of primary lipid oxidation were evaluated using freeze-dried lecithin liposomes as a model membrane system. Ethanol precipitation increased the GOS content from 22.8% to 50.7% (w/w) while reducing the proportion of monosaccharides from 40.0% to 15.5% (w/w). *Tg* was strongly influenced by carbohydrate composition and relative humidity (RH), with GOS-enriched preparations exhibiting higher *Tg* values than the crude reaction mixture. The formation of primary lipid oxidation products, assessed by UV spectroscopy through conjugated diene formation at 234 nm, was significantly lower in liposomes freeze-dried with GOS-rich preparations or sucrose than in phosphate-buffered saline (PBS) controls, particularly under storage conditions in which the carbohydrate matrices remained in the glassy state. At higher RH, transition to the rubbery state was associated with increased conjugated diene formation. These findings suggest that the protective effect of GOS-rich formulations is associated with the physicochemical properties of the carbohydrate matrix and is consistent with the proposed role of the glassy state in limiting molecular mobility, rather than direct antioxidant activity. Beyond their established prebiotic properties, GOS-rich preparations may also contribute to the stability of dehydrated lipid-containing systems, supporting their use as multifunctional food ingredients.

## 1. Introduction

Galacto-oligosaccharides (GOS) are well-established prebiotic carbohydrates with key nutritional benefits due to their ability to selectively stimulate beneficial gut microbiota [1]. They also enhance mineral absorption and provide a low-calorie sweetening effect, further contributing to their nutritional value. From a technological perspective, they are highly valued for their excellent solubility, stability across a wide range of pH and temperature conditions, and favorable textural properties. These characteristics make them particularly suitable for the formulation of functional foods (e.g., infant formulas, dairy products, and baked goods), where they contribute to both enhanced health benefits and improved sensory quality [2].

GOS can be synthesized from lactose via an enzymatic transgalactosylation reaction catalyzed by β-galactosidases [3,4]. β-Galactosidases from microbial sources, such as *Kluyveromyces*, *Aspergillus*, and *Bacillus* species, are commonly employed due to their high enzymatic activity and suitability for industrial-scale processes [5]. The resulting GOS mixture generally consists of β-linked galactose oligomers, which are resistant to digestion in the upper gastrointestinal tract and are selectively fermented by beneficial gut microbiota, thereby conferring their well-established prebiotic properties [6,7,8].

Because the reaction mixture contains GOS together with residual lactose, glucose, and galactose, the removal of mono- and disaccharides is necessary to increase GOS purity and overall yield. GOS purification is typically achieved through a combination of downstream processes aimed at removing low-molecular-weight sugars. Ethanol precipitation is frequently applied as a purification step, substantially increasing GOS purity and reducing the load on eventual membrane filtration or chromatographic processes. This strategy facilitates the production of GOS preparations with high purity, suitable for incorporation into food products and functional ingredients [9,10,11].

Beyond their established nutritional benefits, growing interest has focused on the technological functionality of GOS within complex food matrices, particularly regarding their interactions with macromolecules such as proteins and lipids and their effect on structure and stability. Previous studies have demonstrated that GOS act as effective cryoprotectants for freeze-dried lactic acid bacteria (LAB), as they can replace water molecules, form protective glassy matrices, preserve membrane integrity, and enhance cellular stability during both freeze-drying and subsequent storage [12,13]. GOS protect LAB during drying by stabilizing cell membranes, limiting damage caused by ice crystal formation, and promoting the formation of a glassy matrix, similar to the mechanisms described for other protective sugars such as trehalose. These combined effects contribute to maintain cell viability and functionality, with the protective efficiency being closely associated with the DP and the thermophysical properties of the GOS mixture [14,15].

Lipid oxidation represents a major challenge for phospholipid-based membranes in both biomedical applications and biological systems. It is a complex process involving the sequential formation of primary and secondary oxidation products. Among the available analytical approaches, monitoring the increase in absorbance at 234 nm by UV–vis spectroscopy is widely used to detect the formation of conjugated dienes, which are primary products generated during the early stages of lipid peroxidation. Model membrane systems, such as liposomes employed in drug delivery, closely resemble cytoplasmic membranes in structure and composition, particularly due to their high proportion of unsaturated phospholipids [16]. This compositional feature makes them especially susceptible to oxidative degradation. Oxidative damage to membrane lipids can profoundly affect membrane architecture and function. Lipid peroxidation alters bilayer fluidity, increases permeability, and promotes structural destabilization, ultimately leading to premature drug leakage and reduced therapeutic efficacy in liposomal systems. In living cells, these alterations compromise membrane-associated processes and overall viability. These effects are especially pronounced in freeze-dried bacterial systems, where dehydration and subsequent storage enhance exposure of membrane lipids to oxygen and mechanical stress. This promotes lipid peroxidation, loss of membrane integrity, impaired rehydration capacity, and reduced long-term stability [16,17,18]. Lipid oxidation can be readily monitored by UV–vis spectroscopy through the increase in absorbance at 234 nm, which reflects the formation of conjugated dienes during the early stages of the peroxidation process [19].

Some authors have suggested that certain carbohydrates, including oligosaccharides, may reduce the lipid oxidation. These protective effects are generally attributed to the ability of carbohydrate matrices to preserve membrane structure by reducing molecular mobility and limiting water plasticization during storage, rather than to an intrinsic antioxidant activity [17,20,21,22]. Several studies have suggested that sugars may contribute to the stability of dehydrated biological systems by modifying the physicochemical properties of the surrounding matrix, including water availability, molecular mobility, and membrane organization [23,24]. However, despite the widespread use of GOS as functional food ingredients, little information is available regarding the influence of GOS composition on the lipid oxidation in freeze-dried lipid-based systems during storage. In particular, the relationship between GOS composition, the physical state of the matrix, and lipid oxidation has not been systematically investigated in freeze-dried model membranes. In this context, the aim of this work was to evaluate how differences in the composition of two GOS preparations obtained by enzymatic synthesis and ethanol purification affect the physicochemical properties of freeze-dried lecithin liposomes and the formation of primary lipid oxidation products during storage under different relative humidity conditions. Conjugated diene formation was used as an indicator of the early stages of lipid peroxidation in this model membrane system. A better understanding of the relationship between GOS composition, the physicochemical properties of dried matrices, and lipid stability may contribute to the rational development of multifunctional carbohydrate-based ingredients for food and biological applications.

## 2. Materials and Methods

### 2.1. Preparation of GOS Samples

#### 2.1.1. Enzymatic Synthesis

GOS were synthesized from a supersaturated suspension of lactose monohydrate (Cicarelli, Santa Fe, Argentina) (40% w/v, pH 4.5) via enzymatic transgalactosylation using galadosidase from Aspergillus oryzae (Biolactase F Conc, BIOCON S.a., Barcelona, Spain). The enzyme-to-lactose ratio was maintained constant in all reactions at 50 IU/g of lactose. Reactions were carried out at 40 °C under constant stirring (100 rpm) [4,25]. The reaction was allowed to proceed for 8 h to favor lactose hydrolysis and transgalactosylation. The reaction was stopped by heating the mixture to 85–95 °C for 10 min. Then, the reaction mixture was clarified by centrifugation (10,000× *g*, 10 min) to remove denatured protein and insoluble material. The resulting mixture containing GOS (GOS R) was frozen at −20 °C until further use.

#### 2.1.2. Ethanol Purification of GOS

Ethanol precipitation was applied to increase the proportion of GOS in the mixture by partially removing mono- and disaccharides, following a modified version of the method described by Sen et al. (2011) [10]. Briefly, a 20 mL aliquot of GOS R was mixed with 280 mL of 96% (v/v) ethanol (Pharmacopeia Grade, Bioalcohol, Porta S.A., Córdoba, Argentina), resulting in a final sugar concentration of 27 g/L and an ethanol concentration of 89.6% (v/v). The solutions were incubated at 37 °C for 16 h to promote the precipitation of higher molecular weight saccharides. The precipitated fraction was separated by decantation, and ethanol was removed by evaporation at 37 °C. The recovered solid was then re-dissolved in water to obtain a solution of purified GOS (GOS P), containing 20 g of sugars/100 mL and stored at −20 °C until further use.

#### 2.1.3. Carbohydrate Composition of the Products

The compositions of the GOS R, GOS P and the supernatant after ethanol purification were analyzed by High-Performance Liquid Chromatography (HPLC) on a Perkin-Elmer Series 200 instrument (Perkin-Elmer, Boston, MA, USA) equipped with a refractive index detector and autosampler. A commercial mixture of Vivinal^®^ GOS (Friesland Foods, Zwolle, The Netherlands) was used as a control. All samples were diluted in milli-Q^®^ water (Millipore, Darmstadt, Germany) to concentrations of approximately 10% (w/v) and filtered through 0.22 μm membranes (Millipore Durapore, Billerica, MA, USA). For chromatographic separation, a XBRIDGE AMIDE column (Waters, Benson Polymer, Reno, NV, USA) was used. The column and detector temperatures were maintained at 35 and 40 °C, respectively. Elution was performed using an acetonitrile (HPLC grade, Biopack, Buenos Aires, Argentina)–distilled water solution (75:25) with 0.03% triethylamine (mobile phase) at a flow rate of 0.5 mL/min. External standards of glucose, lactose and galactose (Becton, Dickinson & Co. New York, USA) were included to determine the retention times of each compound present in the reaction products. Chromatograms were integrated using Origin Pro^®^ Graphing & Analysis software (version 2019b, Origin Lab Corporation, Northampton, MA, USA). The composition of monosaccharides (glucose + galactose) and lactose in the samples was determined using calibration curves prepared with appropriate standards, assuming that the area of each chromatographic peak is proportional to the weight percentage of the corresponding sugar [26]. The GOS profile was analyzed by comparison with a commercial GOS preparation (Vivinal^®^, Friesland Campina Ingredients, Amersfoort, The Netherlands), previously characterized in terms of degree of polymerization (DP) distribution [14]. Briefly, the carbohydrate composition was determined by HPLC using a PerkinElmer Series 200 system equipped with a refractive index detector and an autosampler. Separation was performed on a BP-100 Ag^+^ column (300 × 4.6 mm; Benson Polymeric Inc., Reno, NV, USA), and data were acquired using TotalChrom software (version 6.3.1). The column and detector temperatures were maintained at 85 °C and 30 °C, respectively. Milli-Q water was used as the mobile phase at a flow rate of 0.5 mL/min. The retention times of the major oligosaccharide fractions (DP3, DP4 and DP5+) were assigned using this reference, and their relative proportions were estimated assuming that the chromatographic peak area is proportional to the mass fraction of each oligosaccharide species. The validity of this approach was verified by material balance, and the carbohydrate composition was expressed as percentage (w/w) of total carbohydrates.

### 2.2. Determination of Glass Transition Temperatures (Tg)

Solutions of GOS R and GOS P 20% (w/v) were lyophilized for 48 h (Heto FD4 apparatus, Denmark; condenser temperature: −50 °C; pressure chamber: 0.04 mBar), and the resulting samples were equilibrated at 20 °C for 14 days in atmospheres of the following saturated salts—LiCl, KCH_3_COO, MgCl_2_, and Mg(NO_3_)_2_ (Analytical grade, Anhedra, Argentina)—achieving relative humidities (RH) of 11%, 22%, 33%, and 52%, respectively. The equilibrated samples were analyzed by Differential Scanning Calorimetry (DSC) using a calorimeter (Model Q100, TA Instruments, New Castle, DE, USA) calibrated with indium, lead, and zinc. Approximately 10–15 mg of each sample was placed in aluminum capsules (TA, New Castle, DE, USA) and hermetically sealed, using an empty capsule as a reference. Glass transition temperatures (*Tg*) were determined using a thermal cycle designed to eliminate the sample’s thermal history and ensure accurate measurement. Samples were first equilibrated at −60 °C and held isothermally for 3 min. They were then heated to 75 °C at 10 °C/min and maintained at this temperature for 3 min, followed by cooling back to −60 °C at the same rate. After a second 3 min isothermal step at −60 °C, samples were reheated to 75 °C at 10 °C/min, and *Tg* was determined from this second heating scan. Thermograms were analyzed using TA Universal Analysis 2000 software (Advantage Software v5.5.24, Newcastle, DE, USA).

### 2.3. Preparation of Liposomes

Liposomes were prepared by a reverse-phase evaporation procedure [27]. An aliquot (0.155 mmol/mL in chloroform -Biopack, Buenos Aires, Argentina) of soy lecithin (Calbiochem, Merck KGaA, Darmstadt, Germany) was dried under a stream of nitrogen. The lipid film was thoroughly dried to remove any chloroform residue. The lipid films were rehydrated with aqueous solutions containing GOS P, GOS R, sucrose or PBS (control). All sugars were at concentrations of 20% (w/v), with a final lipid concentration of 0.039 mmol/mL. PBS composition: KH_2_PO_4_ 0.24 g/L, NaCl 8 g/L, Na_2_HPO_4_ 1.44 g/L, pH 7.2. All salts were of analytical grade and purchased from Anhedra, Buenos Aires, Argentina. The suspensions were heated to 40–50 °C in a water bath with agitation to promote liposome formation.

### 2.4. Storage of Freeze-Dried Liposomes Under Controlled Relative Humidity

To evaluate liposome peroxidation in the presence of different sugar matrices, the suspensions were freeze-dried and subsequently stored under controlled RH (%) at 4 and 37° C. Aliquots of 0.1 mL of liposomes prepared in PBS, GOS R, GOS P and sucrose were fractionated into 1.5 mL tubes, frozen at −80 °C for 24 h, and freeze-dried for 48 h (Heto FD4 apparatus, Heto Lab Equipment, Allerød, Denmark; condenser temperature: −50 °C; pressure chamber: 0.04 mBar). Freeze-dried liposomes were stored for 28 days at 4 °C and 37 °C in sealed atmospheres containing saturated salt solutions of LiCl, MgCl_2_, and NaCl, providing RH of 11%, 33% and 75%, respectively. On days 14 and 28, the samples were removed from the chambers and rehydrated in 0.1 mL of distilled water, at room temperature for 15 min, to assess the extent of peroxidation.

### 2.5. Lipid Peroxidation

**Scheme 1:**
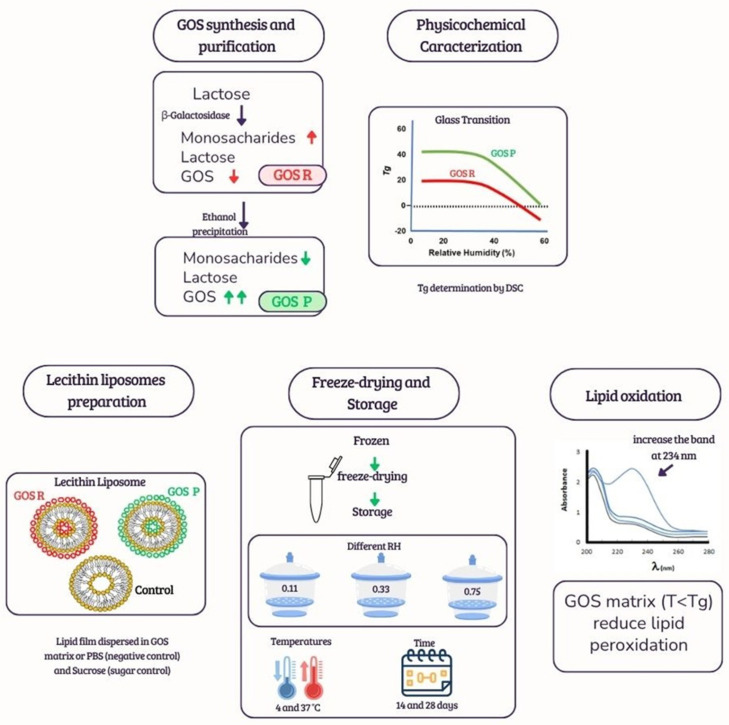
Experimental workflow for evaluating the influence of GOS composition and glass transition behavior on the storage stability of freeze-dried lecithin liposomes.

Lipid peroxidation was evaluated by UV spectroscopy following the approach proposed by Faroux et al. (2021) [19], based on the spectral changes associated with the formation of conjugated dienes during the early stages of lipid oxidation. For this purpose, absorption spectra were recorded between 200 and 300 nm using a microplate reader (Bio-Tek Instruments, Winooski, VT, USA). For spectral acquisition, 10 μL of each liposome suspension was diluted in 1 mL of 96% (v/v) ethanol. The control spectrum of the lipid before freeze-drying showed an exponentially increasing absorbance with a shoulder at approximately 210 nm, corresponding to lipid bonding. Upon exposure of the lipids to oxidative conditions, a characteristic absorption peak appeared at 234 nm, indicating the formation of conjugated dienes. To quantify the specific contribution of this peak while eliminating the intrinsic spectral contribution of each formulation, the spectrum recorded immediately after freeze-drying was used as the baseline and subtracted from the spectra obtained during storage prior to calculating the area under the absorbance curve at 234 nm. Lipid oxidation was then quantified from the integrated area of the corrected spectral band centered at 234 nm (conjugated dienes, primary lipid oxidation products), as described by Faroux et al. (2021) [19], using Prism 8 software (GraphPad Software, Boston, MA, USA). In addition, spectra were recorded immediately before and after freeze-drying to verify that the drying process itself did not induce detectable lipid oxidation. A scheme of the experimental protocol is shown below (Scheme 1).

### 2.6. Statistical Analysis

**Figure 1:**
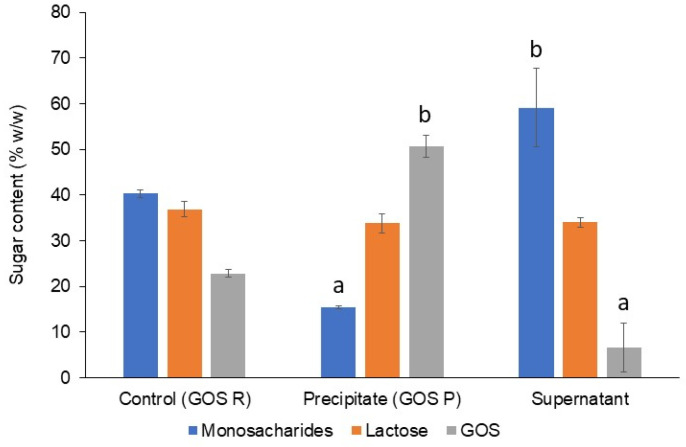
Sugar composition (% w/w) of the control GOS reaction mixture and the fractions obtained after ethanol precipitation (precipitate and supernatant). Control corresponds to mix of reaction (GOS R) after 8 h of incubation. The relative proportions of monosaccharides (glucose + galactose) (blue bars), lactose (orange bars), and total GOS (grey bars) are shown for each sample. Data are expressed as mean ± standard deviation. Different letters indicate statistically significant changes relative to the control (a, decrease; b, increase; one-way ANOVA followed by a two-sided Dunnett’s multiple comparisons test, *p* < 0.05).

All experiments were performed in duplicate using three independent sample preparations, and the results are presented as mean ± standard deviation. Statistical analyses were performed using one-way analysis of variance (ANOVA) using the statistical software STATISTIX 8 (Analytical Software, Tallahassee, FL, USA). For the sugar composition data (Figure 1), Dunnett’s multiple comparison test was used to compare the precipitate and supernatant fractions with the control reaction mixture. For the oxidative stability data, Tukey’s multiple comparison test was applied. Differences were considered significant at *p* < 0.05.

## 3. Results and Discussion

### 3.1. Composition of GOS Preparation

In this study, two GOS preparations were compared: the crude reaction mixture obtained after enzymatic synthesis (GOS R), and the fraction recovered after ethanol precipitation (GOS P). After 8 h of reaction, the sugar profile reached a steady state, with the mixture composed of 40 ± 1% (w/w) monosaccharides (glucose + galactose), 36.9 ± 1.7% (w/w) residual lactose, and 22.8 ± 0.9% (w/w) GOS of different DP (Figure 1, control - GOS R-). The GOS fraction consisted predominantly of DP3 oligosaccharides (63.9%), followed by DP4 (25.9%) and DP5+ (10.2%), as estimated from comparison with a commercial GOS reference. Ethanol precipitation markedly modified the carbohydrate profile. In the precipitated fraction (GOS P), the relative GOS content increased from 22.8% to 50.7 ± 2.3% (w/w), demonstrating substantially increases in the relative proportion of GOS fraction. This enrichment was achieved with an overall solid recovery of ~30% relative to the initial dry matter of GOS R, indicating selective precipitation of higher-DP carbohydrates and limited co-precipitation of low-molecular-weight sugars. The GOS fraction in the precipitate consisted predominantly of DP3 oligosaccharides (57.6%), followed by DP4 (29.4%) and DP5+ (12.5%). The relative distribution of GOS species did not differ significantly from that of the sample before ethanol precipitation (*p* > 0.05). In parallel, monosaccharides were substantially reduced in the precipitate (from ~40% to 15.5 ± 0.4% (w/w)), which reflects their high solubility in aqueous ethanol and preferential partitioning into the supernatant. In contrast, lactose was only partially removed, as its content in the precipitated fraction decreased slightly from 36.9% to 33 ± 1% (w/w). This behavior is consistent with the intermediate solubility of lactose under the applied conditions, leading to partial retention in the solid phase.

The supernatant showed the complementary carbohydrate profile, being composed predominantly of low-molecular-weight sugars. Monosaccharides represented the dominant fraction (59 ± 7% w/w), while lactose accounted for 34% (w/w), confirming their preferential solubilization in ethanol. In contrast, GOS content in the supernatant was markedly reduced (6.6 ± 0.5% w/w), indicating limited solubility of higher-DP oligosaccharides and supporting the use of ethanol precipitation as a simple strategy to increase the relative proportion of GOS in the recovered fraction.

### 3.2. Glass Transition Behavior of GOS Preparation

The *Tg* of a given compound is a determining factor for its incorporation into food product formulations, as it governs physical stability, molecular mobility, and resistance to structural collapse during processing and storage. In amorphous carbohydrate systems, *Tg* is strongly influenced by compositional factors, particularly purity and the distribution of molecular weights. Water acts as a powerful plasticizer in carbohydrate matrices, promoting the transition from a glassy, rigid state to a rubbery, more mobile state [28].

**Figure 2:**
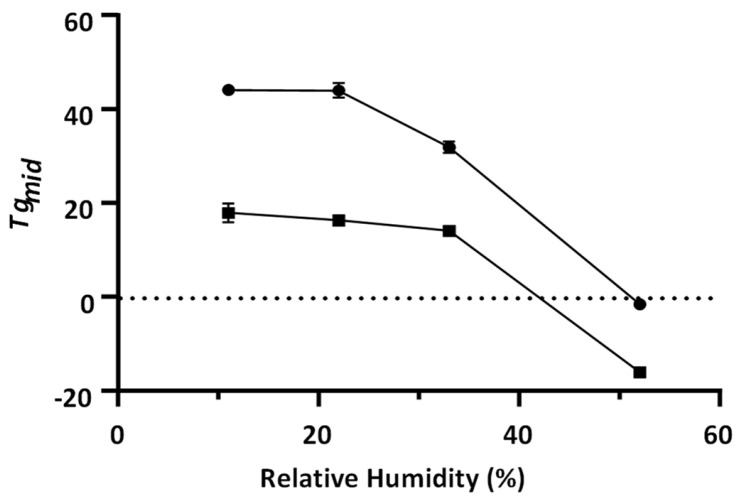
*Tg* of purified GOS (GOS P, ●) and crude GOS (GOS R, ■) as a function of RH (%). Samples were equilibrated at controlled RH conditions. Data are expressed as mean ± standard deviation.

Figure 2 shows the *Tg* of the two GOS mixtures (GOS P and GOS R) as a function of the RH, indicating a strong influence of both carbohydrate composition and RH on *Tg* values [29,30]. For a given RH, GOS R consistently exhibited lower *Tg* values than GOS P, with differences of approximately 20 °C. This behavior can be attributed to the higher proportion of monosaccharides in GOS R (Figure 1), which act as low-molecular-weight plasticizers, increasing molecular mobility and reducing the temperature at which the glass–rubber transition occurs [30]. This compositional effect was maintained across all RH levels tested. These findings reinforce that molecular weight distribution is a key determinant of thermophysical stability, not only in bulk carbohydrate matrices but also in membrane-associated systems. The enrichment in higher-DP fractions in GOS P (Figure 1) reduces the plasticizing effect of low-molecular-weight sugars and broadens the temperature range over which the system remains in a glassy state (Figure 2).

Under low RH conditions, *Tg* values found for GOS P ranged between 31 and 44 °C, whereas GOS R exhibited *Tg* values between 14 and 18 °C (Figure 2). These results are consistent with the trends reported by Lans & Vodovotz (2018) [29], who evaluated commercial GOS preparations of different purities [56.5% (w/w)—Oligomate 55NP; Yakult Co., Tokyo, Japan—and 90% (w/w)—Purine GOS; GTC Nutrition, Golden, CO-]. They observed that higher-purity GOS exhibited substantially higher *Tg* values (up to ~70 °C), whereas lower-purity samples showed maximum *Tg* values around 45 °C at low RH. Together, these results reinforce the importance of carbohydrate composition, particularly the relative proportion of low-molecular-weight sugars, in determining the thermophysical behavior of GOS preparations.

This glass–rubber transition has direct implications for physicochemical stability, including susceptibility to crystallization, stickiness, and chemical degradation reactions. Therefore, differences in GOS purity and composition may modulate water sensitivity and thermal behavior, ultimately influencing their performance in food and bioproduct applications [29].

Although *Tg* and membrane phase transition temperature (Tm) describe different physicochemical phenomena, both parameters are influenced by carbohydrate composition and molecular mobility. Previous FTIR studies have shown that higher-DP GOS increase the Tm of model membranes through interfacial interactions without penetrating the hydrophobic core [31]. Together with the present *Tg* results, these observations suggest that enrichment in higher-DP GOS may contribute to improved structural stability of dehydrated lipid-containing systems.

### 3.3. Effect of GOS Matrices on Lipid Oxidation During Storage

**Figure 3:**
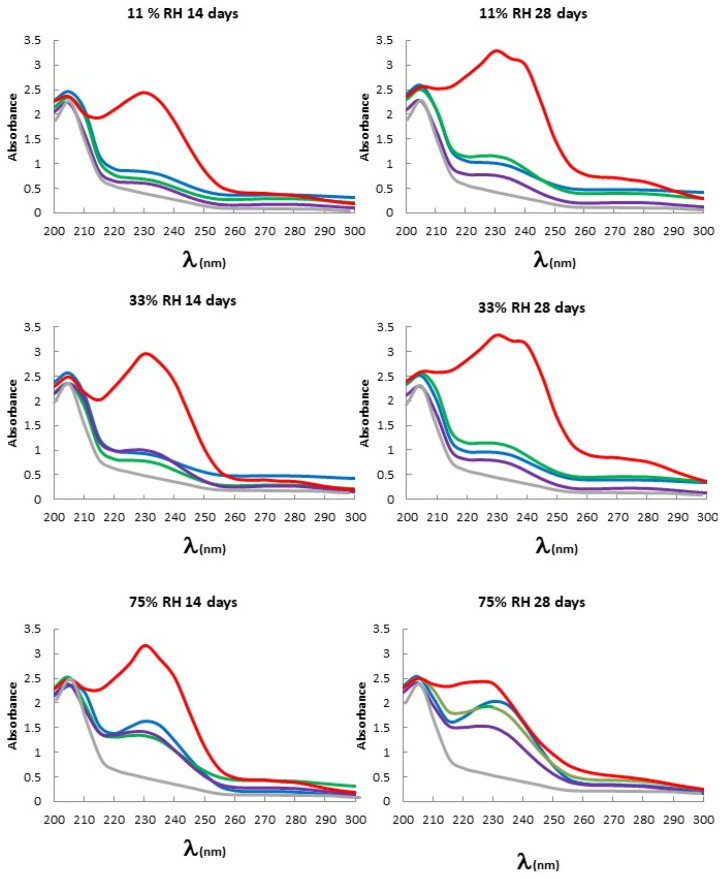
UV absorption spectra (200–300 nm) of freeze-dried lecithin liposomes formulated with purified GOS (GOS P, blue spectra), reaction mixture GOS (GOS R, violet spectra), sucrose (SUC, green spectra), or PBS (control, red spectra), after storage at 4 °C under different RH conditions (11%, 33%, and 75%) for 14 and 28 days. The spectra of liposomes prepared in PBS before freeze-drying are shown in gray.

Figure 3 shows the UV absorption spectra (200–300 nm) of freeze-dried lecithin liposomes after storage at 4 °C under different RH conditions for 14 and 28 days. The UV-based method employed in this study provides a rapid assessment of the formation of primary lipid oxidation products through spectral analysis of conjugated dienes. Although it does not quantify secondary oxidation products, this approach has been previously validated for monitoring oxidative changes in lecithin liposomes and was considered appropriate for comparing the oxidative stability of the different carbohydrate formulations [19]. The control samples (freeze-dried with PBS, red spectra) exhibited a pronounced increase in the AUC at 234 nm, indicating increased formation of primary lipid oxidation products. In contrast, liposomes freeze-dried in the presence of sugars (GOS P -blue-, GOS R -violet-, or sucrose -green-) displayed markedly lower absorbance at this wavelength, indicating improved oxidative stability compared with PBS. Although a slight increase in AUC was observed after 28 days, this effect was more evident at higher RH levels. Sucrose was included in this study as a reference lyoprotectant because it is one of the most widely used and extensively characterized carbohydrates for the stabilization of freeze-dried liposomal formulations. Therefore, the oxidative stability achieved with the GOS preparations was evaluated relative to this conventional benchmark, allowing the protective performance of the synthesized GOS to be assessed against a well-established formulation.

Samples stored at 37 °C rapidly underwent structural collapse of the freeze-dried matrix, accompanied by visible browning, consistent with extensive oxidative deterioration. Under these conditions, the UV spectra were no longer suitable for comparing lipid oxidation among formulations, since advanced oxidation is associated with the degradation of conjugated dienes and the formation of secondary oxidation products, resulting in lower absorbance values despite greater oxidative damage [32]. This observation highlights the importance of considering both storage temperature and relative humidity for stability of dehydrated lipid-containing systems. At this temperature, the *Tg* of most carbohydrate matrices was below or nearly the same as the storage temperature of all samples conditions, resulting in the transition to the rubbery state, and favoring the formation of lipid oxidation products. Consequently, meaningful comparisons among formulations could not be performed, and these samples were not included in the comparative analysis.

**Figure 4:**
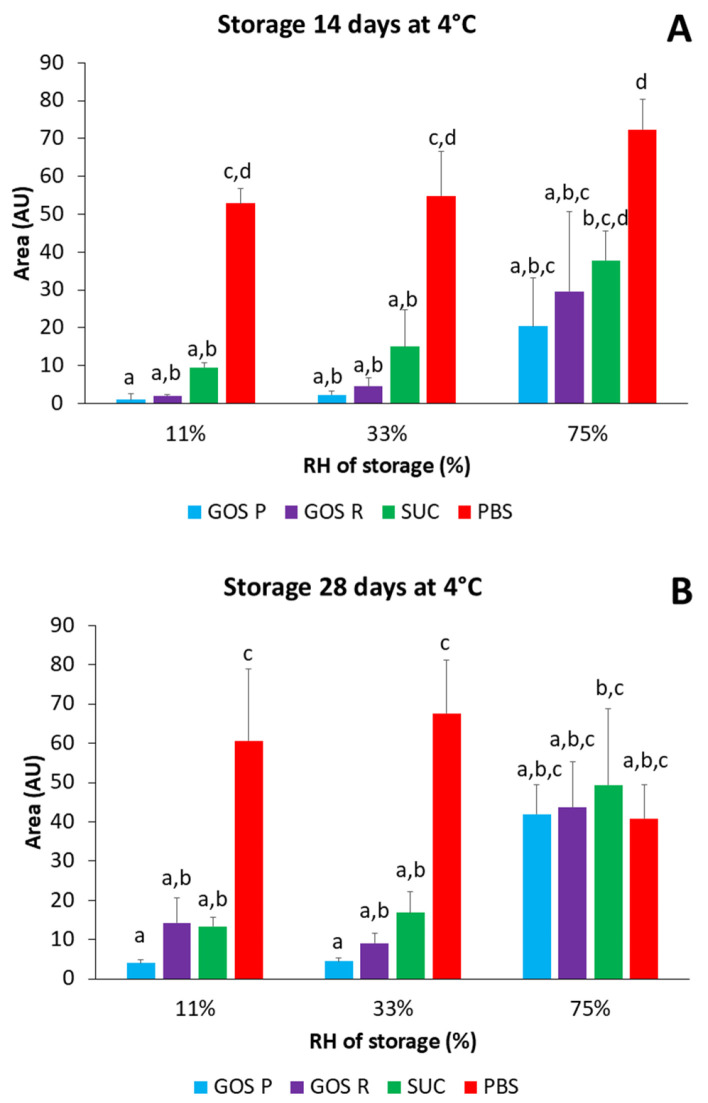
Lipid peroxidation of freeze-dried lecithin liposomes stored at 4 °C for 14 (**A**) and 28 (**B**) days under different RH conditions (11%, 33%, and 75%). Peroxidation was quantified as the area under the curve (AUC) at 234 nm. Liposomes were formulated with purified GOS (GOS P, blue bars), reaction mixture GOS (GOS R, violet bars), sucrose (SUC, green bars), or phosphate-buffered saline (PBS, control, red bars). Data are expressed as median ± standard deviation. Different lowercase letters indicate statistically significant differences among treatments within each storage condition (ANOVA followed by Tukey’s test, *p* < 0.05).

To facilitate quantitative comparison among treatments and storage conditions, the AUC at 234 nm was calculated (Figure 4). After 14 days of storage, significant lower peroxidation indexes were observed in all sugar-containing samples compared to PBS, supporting the protective role of carbohydrate matrices. Notably, at 11% RH, GOS P (blue bars) showed the lowest peroxidation index at both 14 and 28 days, suggesting superior stabilization under low-moisture conditions. At 75% RH, however, a progressive increase in the area at 234 nm was detected after 14 days, particularly for sugar-containing samples. Under these conditions, the *Tg* of the sugar matrices was below the storage temperature (4 °C) (Figure 2), resulting in a transition to the rubbery state. This interpretation is consistent with previous studies reporting enhanced oxygen diffusion and faster lipid oxidation in carbohydrate matrices above their glass transition temperature [33]. These results demonstrate that oxidative stability in freeze-dried liposomal systems is primarily governed by the physical state of the carbohydrate matrix rather than by classical antioxidant mechanisms.

After 28 days, samples stored at 11% and 33% RH, where *Tg* remained above the storage temperature, did not exhibit significant increases in peroxidation compared to 14 days (*p* > 0.05), indicating that lipid oxidation remained relatively stable under conditions where the carbohydrate matrices were maintained in the glassy state. In contrast, samples stored at 75% RH exhibited structural collapse associated with the rubbery state. Concomitant changes in the UV spectral profile were also observed, likely resulting from impaired liposome rehydration and incomplete dispersion, together with the progression of lipid oxidation beyond the stage of conjugated diene formation. As oxidation advances, conjugated dienes are progressively degraded into secondary oxidation products, leading to reduced absorbance at 234 nm despite more extensive lipid deterioration [32].

These results highlight the close relationship between the physical state of the carbohydrate matrix, determined by *Tg* and storage RH, and the oxidative stability of freeze-dried liposomal systems. This interpretation is consistent with previous studies describing reduced molecular mobility in glassy carbohydrate matrices.

The comparable protective behavior of GOS-rich formulations and sucrose suggests that preservation of the glassy state, rather than the specific identity of the carbohydrate, is a major determinant of oxidative stability under the conditions evaluated. According to previous studies, maintenance of the glassy state may reduce molecular mobility, modify the hydration environment of phospholipids, and thereby slow oxygen diffusion and lipid peroxidation [33,34]. These mechanisms were not directly evaluated in the present study and therefore should be regarded as plausible explanations rather than demonstrated effects. Nevertheless, the results suggest the potential use of GOS-rich matrices as protective excipients in freeze-dried lipid-based systems.

Taken together, the present results indicate that GOS-rich preparations improve the oxidative stability of freeze-dried liposomal systems compared with sugar-free controls, with a protective effect comparable to that of sucrose under the same storage conditions. The observed behavior was closely associated with the physical state of the carbohydrate matrix, highlighting the importance of maintaining the system below its *Tg* to preserve oxidative stability during storage. Although the mechanisms responsible for this effect were not directly investigated, the results are consistent with previous studies relating glassy-state matrices to reduced molecular mobility and slower deterioration reactions. Beyond their well-established prebiotic properties, these findings suggest that GOS-rich preparations may also provide technological functionality as protective carbohydrate matrices in dehydrated lipid-containing systems, supporting their potential application in functional food formulations where both nutritional value and storage stability are desirable.

## 4. Conclusions

The present study showed that GOS-rich preparations reduce the formation of primary lipid oxidation products in freeze-dried lecithin liposomes compared with the sugar-free control. Ethanol precipitation increased the relative GOS content while reducing the proportion of low-molecular-weight sugars, resulting in higher glass transition temperatures (*Tg*) and enhanced thermophysical stability of the carbohydrate matrices.

The formation of primary lipid oxidation was lower under storage conditions where the carbohydrate matrices remained in the glassy state (Tstorage < *Tg*), whereas conditions favoring the rubbery state were associated with increased oxidative deterioration. These observations support the hypothesis that the protective effect of GOS-rich preparations is primarily related to the physicochemical properties of the carbohydrate matrix rather than to a specific antioxidant activity.

Although the underlying mechanisms were not directly investigated, the results are consistent with previous studies proposing that glassy carbohydrate matrices reduce molecular mobility, thereby delaying the initiation of lipid peroxidation. Accordingly, the improved oxidative stability observed in the GOS-rich preparations is consistent with the formation of a more stable glassy matrix. These findings highlight that, in addition to their established prebiotic properties, GOS-rich preparations may also contribute to the stabilization of dehydrated lipid-containing systems through their physicochemical characteristics, thereby combining nutritional value with technological functionality.

## Data Availability

The original contributions presented in this study are included in the article. Further inquiries can be directed to the corresponding author.

## Abbreviations

The following abbreviations are used in this manuscript:

AUC	Area under the curve
DP	Degree of polymerization
DSC	Differential scanning calorimetry
GOS	Galacto-oligosaccharides
GOS P	Purified galacto-oligosaccharides
GOS R	Galacto-oligosaccharide reaction mixture
PBS	Phosphate-buffered saline
RH	Relative humidity
*Tg*	Glass transition temperature
